# Crystal structure of methyl (2*R*,3*S*)-3-[(*tert*-butyl­sulfin­yl)amino]-2-fluoro-3-phenyl­propano­ate

**DOI:** 10.1107/S2056989015023580

**Published:** 2015-12-16

**Authors:** Zhiwei Zhao, Wenqiang Fan, Yixiang Zhang, Ya Li

**Affiliations:** a Department of Chemistry and Chemical Engineering, Shanghai University of Engineering Science, 333 Longteng Road, Shanghai, People’s Republic of China

**Keywords:** crystal structure, fluorine, amino acid, sulfoxide, N—H⋯O hydrogen bonding

## Abstract

The title compound, C_14_H_20_FNO_3_S, contains two chiral carbon centres and the absolute configuration has been confirmed as (2*R*,3*S*). In the crystal, adjacent mol­ecules are linked by weak C—H⋯O hydrogen bonds, generating zigzag chains along the *a*-axis direction.

## Related literature   

For the use of of fluorinated β-amino acids in organic synthesis, see: Marsh (2014 ▸); Niemz & Tirrell (2001 ▸); Chiu *et al.* (2006 ▸). For their synthesis, see: Shang *et al.* (2015 ▸); Yoshinari *et al.* (2011 ▸); Duggan *et al.* (2010 ▸); Peddie & Abell (2012 ▸); Jing *et al.* (2011 ▸); Pan *et al.* (2010 ▸).
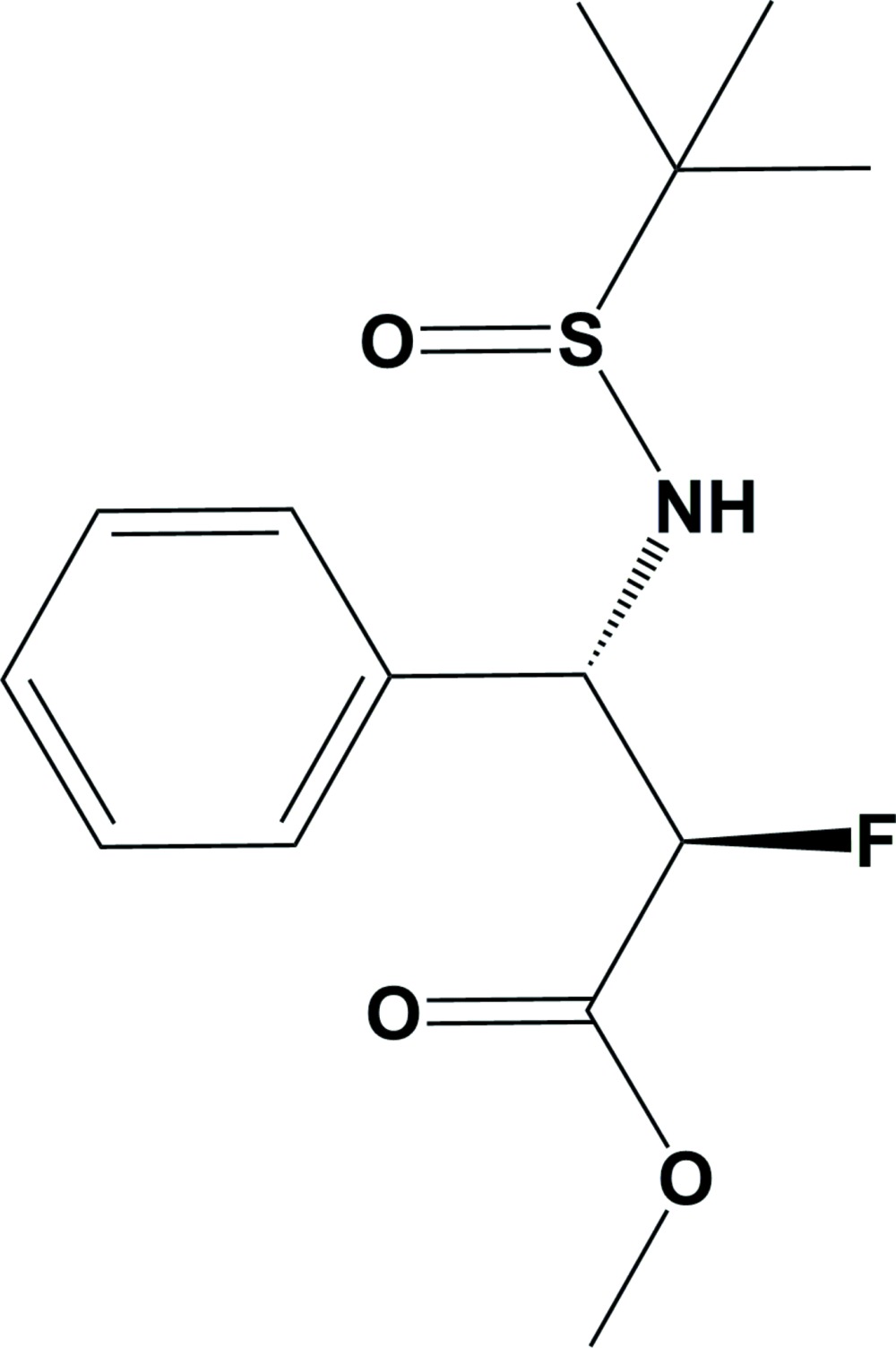


## Experimental   

### Crystal data  

C_14_H_20_FNO_3_S
*M* *_r_* = 301.37
Orthorhombic, 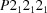

*a* = 9.1809 (14) Å
*b* = 9.2384 (15) Å
*c* = 18.577 (3) Å
*V* = 1575.7 (4) Å^3^
*Z* = 4
Mo *K*α radiation
μ = 0.22 mm^−1^
*T* = 296 K
0.13 × 0.11 × 0.07 mm

### Data collection  

Bruker APEXII CCD diffractometer
Absorption correction: multi-scan (*SADABS*; Bruker, 2007 ▸) *T*_min_ = 0.972, *T*_max_ = 0.985
8176 measured reflections
2773 independent reflections
2542 reflections with *I* > 2σ(*I*)
*R*_int_ = 0.022

### Refinement  

*R*[*F* ^2^ > 2σ(*F* ^2^)] = 0.033
*wR*(*F* ^2^) = 0.088
*S* = 1.04
2773 reflections
186 parameters
H-atom parameters constrained
Δρ_max_ = 0.27 e Å^−3^
Δρ_min_ = −0.29 e Å^−3^
Absolute structure: Flack (1983 ▸)
Absolute structure parameter: 0.05 (8)

### 

Data collection: *APEX2* (Bruker, 2007 ▸); cell refinement: *SAINT* (Bruker, 2007 ▸); data reduction: *SAINT*; program(s) used to solve structure: *SHELXS97* (Sheldrick, 2008 ▸); program(s) used to refine structure: *SHELXL97* (Sheldrick, 2008 ▸); molecular graphics: *SHELXTL* (Sheldrick, 2008 ▸) and *PLATON* (Spek, 2009 ▸); software used to prepare material for publication: *SHELXTL*.

## Supplementary Material

Crystal structure: contains datablock(s) I, New_Global_Publ_Block. DOI: 10.1107/S2056989015023580/su5256sup1.cif

Structure factors: contains datablock(s) I. DOI: 10.1107/S2056989015023580/su5256Isup2.hkl

Supporting information file. DOI: 10.1107/S2056989015023580/su5256Isup3.cml

**Fig. 1.**
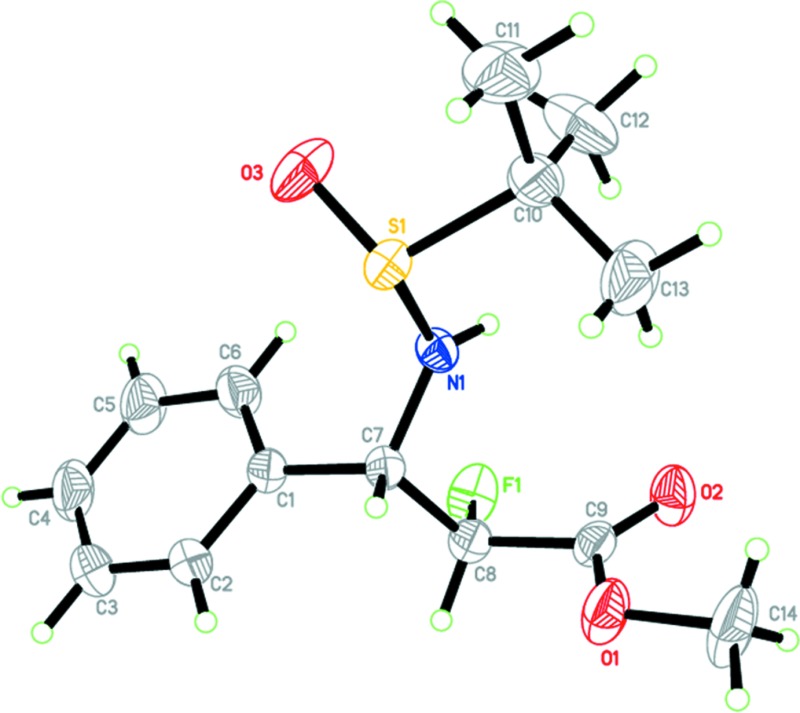
Molecular structure of the title compound, with atom labeling. Displacement ellipsoids are drawn at the 50% probability level.

**Fig. 2.**
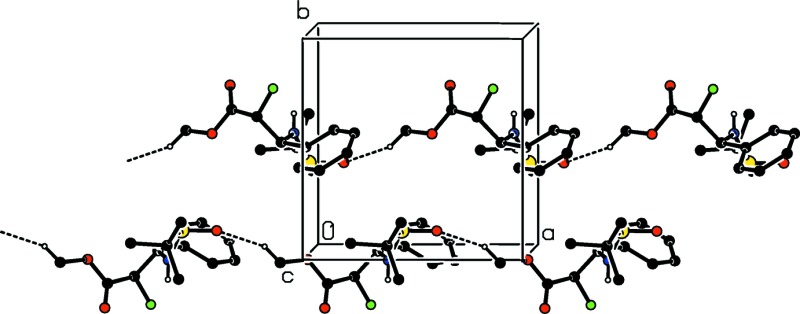
A partial view along the c axis of the crystal packing of the title compound. Hydrogen bonds are shown as dashed lines (see Table 1).

. DOI: 10.1107/S2056989015023580/su5256fig1.tif
Mol­ecular structure of the title compound, with atom labeling. Displacement ellipsoids are drawn at the 50% probability level.

c . DOI: 10.1107/S2056989015023580/su5256fig2.tif
A partial view along the *c* axis of the crystal packing of the title compound. Hydrogen bonds are shown as dashed lines (see Table 1).

CCDC reference: 1441329

Additional supporting information:  crystallographic information; 3D view; checkCIF report

## Figures and Tables

**Table 1:** Hydrogen-bond geometry (Å, °)

*D*—H⋯*A*	*D*—H	H⋯*A*	*D*⋯*A*	*D*—H⋯*A*
C14—H14*B*⋯O3^i^	0.96	2.79	3.045 (4)	135

Symmetry code: (i) 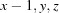.

## supplementary crystallographic information

### S1. Synthesis and crystallization

LiHMDS (1.5 ml, 1.0 mol/l in THF) was added to a solution of methyl fluoro­acetate (138 mg, 1.5 mmol), (Rs)—*N*-benzyl­idene- 2-methyl­propane-2-sulfinamide (209 mg, 1.0 mmol), *N*,*N*,*N*',*N*'-tetra­methyl-ethane-1,2-di­amine (0.3 ml), and THF (3 ml) at 203 K. The reaction mixture was stirred for 30 min, then saturated NH_4_Cl—H_2_O (5 ml) was added, and the quenched reaction mixture was extracted with ethyl acetate (3 × 20 ml). The combined organic layers were dried over anhydrous Na_2_SO_4_. The obtained compound was recrystallized from ethyl acetate/hexane (1:2) to give colorless crystals.

### S1.1. Refinement

Crystal data, data collection and structure refinement details are summarized in Table 2. A l l the H atoms were placed at calculated positions and treated as riding atoms: N—H = 0.86 Å, C—H = 0.93–0.96 Å with *U*_iso_(H) = 1.5*U*_eq_(*C*-methyl and 1.2*U*_eq_(N,*C*) for other H atoms.

### Crystal data

**Table:**

C_14_H_20_FNO_3_S	*F*(000) = 640
*M**_r_* = 301.37	*D*_x_ = 1.270 Mg m^−^^3^
Orthorhombic, *P*2_1_2_1_2_1_	Mo *K*α radiation, λ = 0.71073 Å
Hall symbol: P 2ac 2ab	Cell parameters from 3418 reflections
*a* = 9.1809 (14) Å	θ = 2.2–25.5°
*b* = 9.2384 (15) Å	µ = 0.22 mm^−^^1^
*c* = 18.577 (3) Å	*T* = 296 K
*V* = 1575.7 (4) Å^3^	Block, colorless
*Z* = 4	0.13 × 0.11 × 0.07 mm

### Data collection

**Table:**

Bruker APEXII CCD diffractometer	2773 independent reflections
Radiation source: fine-focus sealed tube	2542 reflections with *I* > 2σ(*I*)
Graphite monochromator	*R*_int_ = 0.022
φ and ω scans	θ_max_ = 25.0°, θ_min_ = 2.2°
Absorption correction: multi-scan (*SADABS*; Bruker, 2007)	*h* = −7→10
*T*_min_ = 0.972, *T*_max_ = 0.985	*k* = −11→11
8176 measured reflections	*l* = −22→21

### Refinement

**Table:**

Refinement on *F*^2^	Hydrogen site location: inferred from neighbouring sites
Least-squares matrix: full	H-atom parameters constrained
*R*[*F*^2^ > 2σ(*F*^2^)] = 0.033	*w* = 1/[σ^2^(*F*_o_^2^) + (0.0505*P*)^2^ + 0.1714*P*] where *P* = (*F*_o_^2^ + 2*F*_c_^2^)/3
*wR*(*F*^2^) = 0.088	(Δ/σ)_max_ < 0.001
*S* = 1.04	Δρ_max_ = 0.27 e Å^−^^3^
2773 reflections	Δρ_min_ = −0.29 e Å^−^^3^
186 parameters	Extinction correction: *SHELXL97* (Sheldrick, 2008), Fc^*^=kFc[1+0.001xFc^2^λ^3^/sin(2θ)]^-1/4^
0 restraints	Extinction coefficient: 0.0117 (16)
Primary atom site location: structure-invariant direct methods	Absolute structure: Flack (1983), ???? Friedel pairs
Secondary atom site location: difference Fourier map	Absolute structure parameter: 0.05 (8)

### Special details

**Table:**

Geometry. All e.s.d.'s (except the e.s.d. in the dihedral angle between two l.s. planes) are estimated using the full covariance matrix. The cell e.s.d.'s are taken into account individually in the estimation of e.s.d.'s in distances, angles and torsion angles; correlations between e.s.d.'s in cell parameters are only used when they are defined by crystal symmetry. An approximate (isotropic) treatment of cell e.s.d.'s is used for estimating e.s.d.'s involving l.s. planes.
Refinement. Refinement of *F*^2^ against ALL reflections. The weighted *R*-factor *wR* and goodness of fit *S* are based on *F*^2^, conventional *R*-factors *R* are based on *F*, with *F* set to zero for negative *F*^2^. The threshold expression of *F*^2^ > σ(*F*^2^) is used only for calculating *R*-factors(gt) *etc*. and is not relevant to the choice of reflections for refinement. *R*-factors based on *F*^2^ are statistically about twice as large as those based on *F*, and *R*- factors based on ALL data will be even larger.

### Fractional atomic coordinates and isotropic or equivalent isotropic displacement parameters (Å^2^)

**Table:**

	*x*	*y*	*z*	*U*_iso_*/*U*_eq_	
S1	0.45246 (5)	0.13512 (6)	1.04644 (3)	0.04566 (16)	
C1	0.4525 (2)	0.0513 (2)	0.86771 (10)	0.0454 (5)	
C2	0.4258 (3)	0.1407 (3)	0.80959 (13)	0.0645 (7)	
H2	0.3412	0.1960	0.8085	0.077*	
C3	0.5233 (3)	0.1489 (4)	0.75313 (14)	0.0799 (8)	
H3	0.5032	0.2089	0.7142	0.096*	
C4	0.6486 (3)	0.0701 (4)	0.75394 (14)	0.0790 (9)	
H4	0.7134	0.0755	0.7156	0.095*	
C5	0.6780 (3)	−0.0166 (3)	0.81134 (15)	0.0763 (8)	
H5	0.7640	−0.0698	0.8124	0.092*	
C6	0.5812 (3)	−0.0262 (3)	0.86807 (13)	0.0614 (6)	
H6	0.6029	−0.0855	0.9070	0.074*	
C7	0.3370 (2)	0.0338 (2)	0.92539 (10)	0.0412 (4)	
H7	0.2837	0.1254	0.9288	0.049*	
C8	0.2285 (2)	−0.0833 (2)	0.90203 (11)	0.0485 (5)	
H8	0.1834	−0.0536	0.8566	0.058*	
C9	0.1100 (2)	−0.1116 (2)	0.95639 (13)	0.0467 (5)	
C10	0.3929 (3)	0.0729 (3)	1.13521 (12)	0.0606 (6)	
C11	0.4604 (4)	0.1814 (4)	1.18744 (15)	0.0919 (10)	
H11A	0.4255	0.1626	1.2352	0.138*	
H11B	0.4337	0.2778	1.1734	0.138*	
H11C	0.5646	0.1720	1.1865	0.138*	
C12	0.4469 (5)	−0.0790 (3)	1.15008 (15)	0.1004 (11)	
H12A	0.5481	−0.0858	1.1376	0.151*	
H12B	0.3920	−0.1468	1.1218	0.151*	
H12C	0.4347	−0.1008	1.2002	0.151*	
C13	0.2279 (3)	0.0833 (4)	1.13551 (16)	0.0914 (10)	
H13A	0.1880	0.0102	1.1045	0.137*	
H13B	0.1989	0.1772	1.1186	0.137*	
H13C	0.1924	0.0692	1.1836	0.137*	
C14	−0.1016 (3)	−0.0130 (4)	1.00859 (19)	0.0961 (11)	
H14A	−0.1445	−0.1076	1.0052	0.144*	
H14B	−0.1733	0.0589	0.9969	0.144*	
H14C	−0.0670	0.0026	1.0567	0.144*	
F1	0.30213 (17)	−0.21153 (14)	0.89103 (8)	0.0696 (4)	
N1	0.38966 (18)	−0.00242 (17)	0.99719 (8)	0.0410 (4)	
H1	0.3884	−0.0897	1.0133	0.049*	
O1	0.02014 (16)	−0.00250 (18)	0.95826 (11)	0.0748 (5)	
O2	0.10015 (19)	−0.21952 (17)	0.99189 (10)	0.0648 (5)	
O3	0.61290 (18)	0.1341 (2)	1.04867 (11)	0.0792 (5)	

### Atomic displacement parameters (Å^2^)

**Table:**

	*U*^11^	*U*^22^	*U*^33^	*U*^12^	*U*^13^	*U*^23^
S1	0.0395 (3)	0.0456 (3)	0.0519 (3)	−0.0028 (2)	0.0025 (2)	−0.0062 (2)
C1	0.0440 (11)	0.0485 (11)	0.0438 (11)	−0.0090 (10)	0.0010 (10)	0.0042 (9)
C2	0.0517 (14)	0.0817 (17)	0.0600 (14)	−0.0088 (13)	−0.0025 (11)	0.0254 (13)
C3	0.0740 (19)	0.111 (2)	0.0545 (14)	−0.0187 (18)	0.0034 (13)	0.0315 (15)
C4	0.0731 (19)	0.101 (2)	0.0632 (17)	−0.0250 (17)	0.0232 (15)	0.0080 (16)
C5	0.0572 (15)	0.0885 (19)	0.0832 (18)	0.0025 (15)	0.0285 (13)	0.0076 (16)
C6	0.0578 (14)	0.0670 (14)	0.0595 (14)	0.0055 (12)	0.0115 (11)	0.0130 (12)
C7	0.0402 (10)	0.0416 (10)	0.0418 (10)	−0.0018 (8)	0.0021 (8)	0.0043 (9)
C8	0.0455 (12)	0.0540 (12)	0.0458 (11)	−0.0051 (10)	0.0000 (9)	0.0011 (10)
C9	0.0369 (10)	0.0445 (11)	0.0588 (12)	−0.0058 (8)	−0.0021 (10)	0.0024 (11)
C10	0.0636 (15)	0.0741 (16)	0.0440 (12)	−0.0009 (13)	0.0036 (11)	−0.0078 (11)
C11	0.094 (2)	0.121 (2)	0.0608 (16)	−0.006 (2)	−0.0073 (16)	−0.0322 (16)
C12	0.152 (3)	0.089 (2)	0.0602 (16)	0.007 (2)	−0.020 (2)	0.0179 (15)
C13	0.0664 (18)	0.133 (3)	0.0744 (18)	−0.0155 (18)	0.0280 (15)	−0.0240 (18)
C14	0.0490 (15)	0.0811 (18)	0.158 (3)	0.0062 (14)	0.0448 (19)	0.019 (2)
F1	0.0689 (9)	0.0569 (8)	0.0828 (10)	−0.0086 (7)	0.0215 (7)	−0.0240 (7)
N1	0.0477 (10)	0.0368 (8)	0.0385 (8)	−0.0010 (7)	0.0006 (7)	0.0035 (7)
O1	0.0440 (9)	0.0610 (10)	0.1193 (15)	0.0081 (8)	0.0214 (10)	0.0283 (10)
O2	0.0621 (10)	0.0489 (9)	0.0835 (12)	−0.0020 (7)	0.0163 (9)	0.0133 (9)
O3	0.0401 (8)	0.0993 (14)	0.0981 (13)	−0.0126 (9)	0.0054 (9)	−0.0301 (12)

### Geometric parameters (Å, º)

**Table:**

S1—O3	1.4737 (17)	C8—H8	0.9800
S1—N1	1.6685 (17)	C9—O2	1.199 (2)
S1—C10	1.830 (2)	C9—O1	1.303 (3)
C1—C2	1.381 (3)	C10—C12	1.513 (4)
C1—C6	1.381 (3)	C10—C13	1.518 (4)
C1—C7	1.516 (3)	C10—C11	1.527 (4)
C2—C3	1.381 (4)	C11—H11A	0.9600
C2—H2	0.9300	C11—H11B	0.9600
C3—C4	1.361 (4)	C11—H11C	0.9600
C3—H3	0.9300	C12—H12A	0.9600
C4—C5	1.361 (4)	C12—H12B	0.9600
C4—H4	0.9300	C12—H12C	0.9600
C5—C6	1.382 (3)	C13—H13A	0.9600
C5—H5	0.9300	C13—H13B	0.9600
C6—H6	0.9300	C13—H13C	0.9600
C7—N1	1.458 (2)	C14—O1	1.460 (3)
C7—C8	1.533 (3)	C14—H14A	0.9600
C7—H7	0.9800	C14—H14B	0.9600
C8—F1	1.379 (3)	C14—H14C	0.9600
C8—C9	1.507 (3)	N1—H1	0.8600
			
O3—S1—N1	110.87 (10)	O1—C9—C8	109.95 (18)
O3—S1—C10	105.74 (12)	C12—C10—C13	112.7 (3)
N1—S1—C10	98.71 (10)	C12—C10—C11	111.1 (2)
C2—C1—C6	117.7 (2)	C13—C10—C11	111.2 (2)
C2—C1—C7	119.5 (2)	C12—C10—S1	111.0 (2)
C6—C1—C7	122.66 (17)	C13—C10—S1	106.35 (19)
C1—C2—C3	120.8 (3)	C11—C10—S1	104.17 (19)
C1—C2—H2	119.6	C10—C11—H11A	109.5
C3—C2—H2	119.6	C10—C11—H11B	109.5
C4—C3—C2	120.6 (3)	H11A—C11—H11B	109.5
C4—C3—H3	119.7	C10—C11—H11C	109.5
C2—C3—H3	119.7	H11A—C11—H11C	109.5
C3—C4—C5	119.4 (2)	H11B—C11—H11C	109.5
C3—C4—H4	120.3	C10—C12—H12A	109.5
C5—C4—H4	120.3	C10—C12—H12B	109.5
C4—C5—C6	120.5 (3)	H12A—C12—H12B	109.5
C4—C5—H5	119.8	C10—C12—H12C	109.5
C6—C5—H5	119.8	H12A—C12—H12C	109.5
C1—C6—C5	120.9 (2)	H12B—C12—H12C	109.5
C1—C6—H6	119.5	C10—C13—H13A	109.5
C5—C6—H6	119.5	C10—C13—H13B	109.5
N1—C7—C1	116.05 (17)	H13A—C13—H13B	109.5
N1—C7—C8	108.22 (16)	C10—C13—H13C	109.5
C1—C7—C8	109.24 (16)	H13A—C13—H13C	109.5
N1—C7—H7	107.7	H13B—C13—H13C	109.5
C1—C7—H7	107.7	O1—C14—H14A	109.5
C8—C7—H7	107.7	O1—C14—H14B	109.5
F1—C8—C9	107.71 (16)	H14A—C14—H14B	109.5
F1—C8—C7	109.26 (18)	O1—C14—H14C	109.5
C9—C8—C7	113.67 (17)	H14A—C14—H14C	109.5
F1—C8—H8	108.7	H14B—C14—H14C	109.5
C9—C8—H8	108.7	C7—N1—S1	116.21 (12)
C7—C8—H8	108.7	C7—N1—H1	121.9
O2—C9—O1	125.5 (2)	S1—N1—H1	121.9
O2—C9—C8	124.6 (2)	C9—O1—C14	116.76 (19)

### Hydrogen-bond geometry (Å, º)

**Table:**

*D*—H···*A*	*D*—H	H···*A*	*D*···*A*	*D*—H···*A*
C14—H14*B*···O3^i^	0.96	2.79	3.045 (4)	135

Symmetry code: (i) *x*−1, *y*, *z*.
