# Supplementary figures and images for: Crystal structure of methyl (2R,3S)-3-[(tert-butyl­sulfin­yl)amino]-2-fluoro-3-phenyl­propano­ate
Source: Acta Crystallogr E Crystallogr Commun. 2015 Dec 16;71(Pt 12):o1055–6. doi: 10.1107/S2056989015023580 (PMC4719976; doi:10.1107/S2056989015023580)

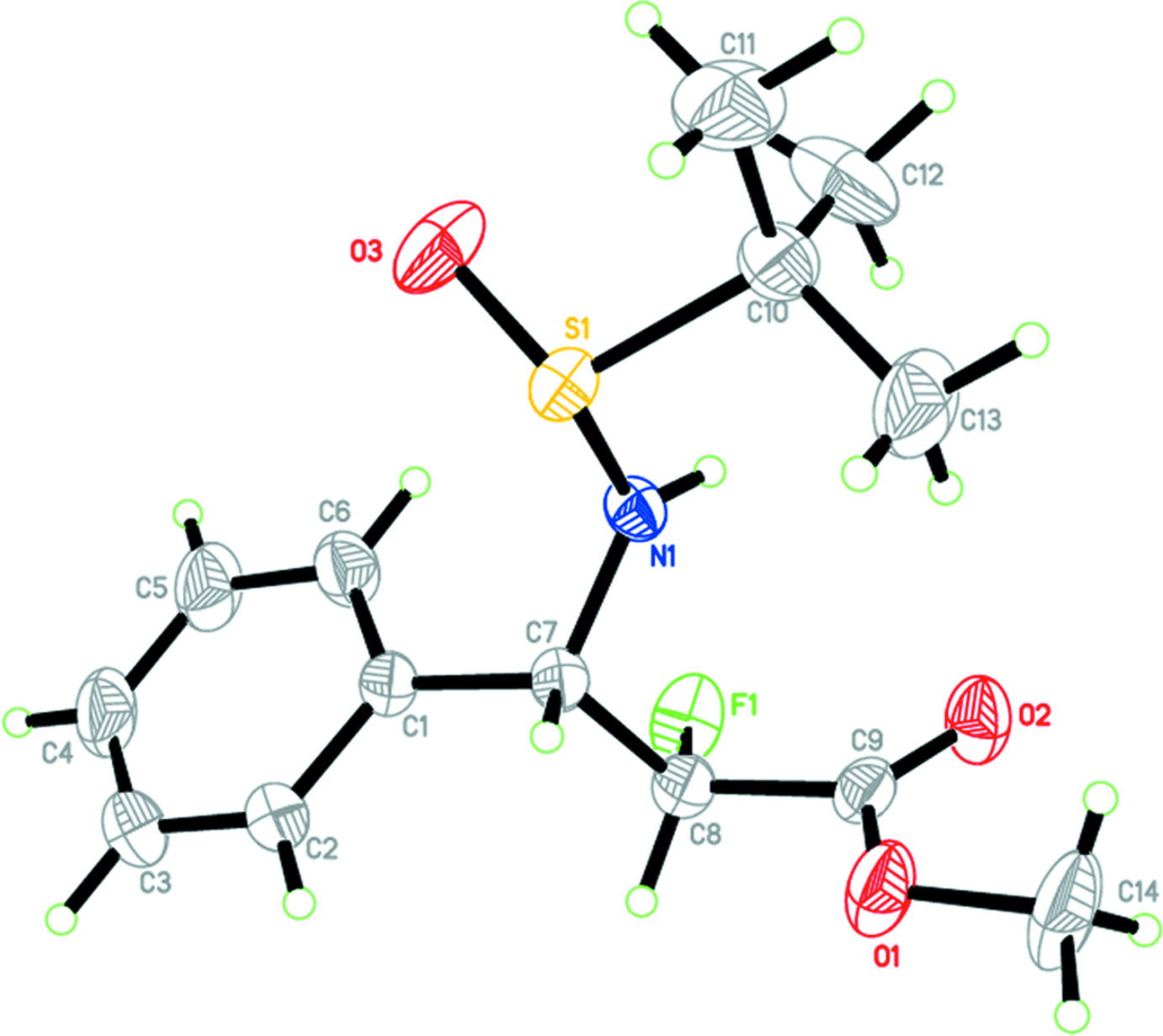

Supplement: Supplementary file 4 [file e-71-o1055-fig1.tif]

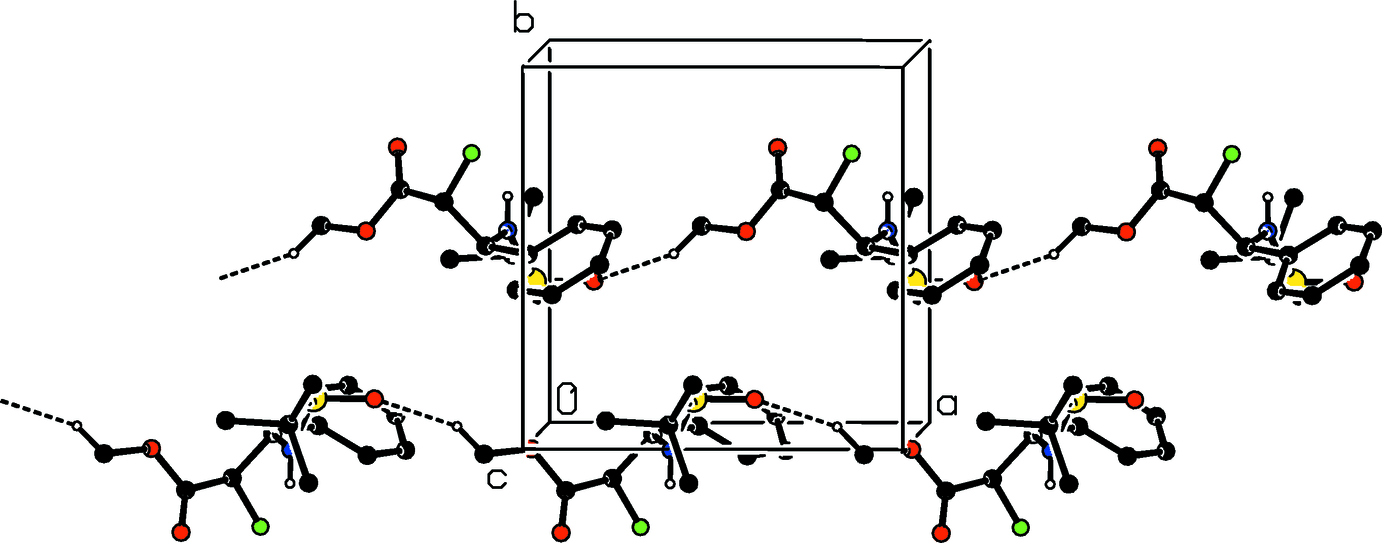

Supplement: Supplementary file 5 [file e-71-o1055-fig2.tif]
